# Membrane estrogen receptor-α levels in MCF-7 breast cancer cells predict cAMP and proliferation responses

**DOI:** 10.1186/bcr958

**Published:** 2004-11-24

**Authors:** Dragoslava Zivadinovic, Bahiru Gametchu, Cheryl S Watson

**Affiliations:** 1 Department of Human Biological Chemistry and Genetics, University of Texas Medical Branch, Galveston, Texas, USA; 2 Department of Pediatrics, Medical College of Wisconsin, Milwaukee, Wisconsin, USA

**Keywords:** adenylyl cyclase, 17β-estradiol, dose response, protein kinase A

## Abstract

**Introduction:**

17β-estradiol (E_2_) can rapidly induce cAMP production, but the conditions under which these cAMP levels are best measured and the signaling pathways responsible for the consequent proliferative effects on breast cancer cells are not fully understood. To help resolve these issues, we compared cAMP mechanistic responses in MCF-7 cell lines selected for low (mER^low^) and high (mER^high^) expression of the membrane form of estrogen receptor (mER)-α, and thus addressed the receptor subform involved in cAMP signaling.

**Methods:**

MCF-7 cells were immunopanned and subsequently separated by fluorescence activated cell sorting into mER^high ^(mER-α-enriched) and mER^low ^(mER-α-depleted) populations. Unique (compared with previously reported) incubation conditions at 4°C were found to be optimal for demonstrating E_2_-induced cAMP production. Time-dependent and dose-dependent effects of E_2 _on cAMP production were determined for both cell subpopulations. The effects of forskolin, 8-CPT cAMP, protein kinase A inhibitor (H-89), and adenylyl cyclase inhibitor (SQ 22,536) on E_2_-induced cell proliferation were assessed using the crystal violet assay.

**Results:**

We demonstrated a rapid and transient cAMP increase after 1 pmol/l E_2 _stimulation in mER^high ^cells; at 4°C these responses were much more reliable and robust than at 37°C (the condition most often used). The loss of cAMP at 37°C was not due to export. 3-Isobutyl-1-methylxanthine (IBMX; 1 mmol/l) only partially preserved cAMP, suggesting that multiple phosphodiesterases modulate its level. The accumulated cAMP was consistently much higher in mER^high ^cells than in mER^low ^cells, implicating mER-α levels in the process. ICI172,780 blocked the E_2_-induced response and 17α-estradiol did not elicit the response, also suggesting activity through an estrogen receptor. E_2 _dose-dependent cAMP production, although biphasic in both cell types, was responsive to 50-fold higher E_2 _concentrations in mER^high ^cells. Proliferation of mER^low ^cells was stimulated over the whole range of E_2_concentrations, whereas the number of mER^high ^cells was greatly decreased at concentrations above 1 nmol/l, suggesting that estrogen over-stimulation can lead to cell death, as has previously been reported, and that mER-α participates. E_2_-mediated activation of adenylyl cyclase and downstream participation of protein kinase A were shown to be involved in these responses.

**Conclusion:**

Rapid mER-α-mediated nongenomic signaling cascades generate cAMP and downstream signaling events, which contribute to the regulation of breast cancer cell number.

## Introduction

It has long been recognized that cAMP is an important intracellular messenger that is capable of regulating diverse functions such as steroidogenesis in the ovary, sugar metabolism in the liver, and contractility of the cardiac muscle [1-5]. In addition to these crucial and specific physiological functions, the cAMP pathway plays an important regulatory role in the development of other complex cellular states such as differentiation, proliferation, and synaptic plasticity. Elevated levels of intracellular cAMP can inhibit the growth of some cell types but stimulate the growth of others. Among the cell types whose growth is stimulated by cAMP are thyroid [6], pituitary [7], and normal human breast epithelial cells in culture [8]. Several studies have demonstrated that cAMP inhibits the growth of established breast cancer cell lines and breast cancer cells in primary culture [8-10]. However, such studies have not completely resolved issues such as the effective levels of cAMP in different cell types, the roles played by other participants in the signaling web that may cooperate to bring about different outcomes, and any subpopulations of response-initiating receptors.

The intracellular cAMP level is determined by the rates of its synthesis and clearance. It is synthesized from ATP via a transmembrane adenylyl cyclase (AC), which is activated by stimulatory G_s _proteins coupled to cell surface receptors (G-protein-coupled receptors). Clearance is regulated either by cyclic nucleotide phosphodiesterases (PDEs) or by an efflux mechanism that secretes or transports cAMP out of cells [11]. The multiplicity of PDEs was recently established [12], but the regulation and utilization of different isoforms are experimental issues that remain unclear.

The influence of 17β-estradiol (E_2_) on MCF-7 breast cancer cell proliferation was previously assigned exclusively to genomic mechanisms. However, it has been known for some time that E_2 _can rapidly (within a few minutes) induce cAMP production, and that this time frame is not compatible with the multiple macromolecular synthetic events necessary for genomic responses [13]. We have an incomplete understanding of the conditions under which cAMP responses are best measured, and of the participation of multiple enzymes in generating and degrading cAMP. There are also reports that some MCF-7 cell sublines either do not respond or do not reliably generate this response (personal communications from many laboratories). Therefore, estrogen receptor (ER)-mediated mechanisms that increase cAMP in MCF-7 and other responsive cell types have not fully been explained. Here, we compare cAMP responses in cell lines selected for low and high expression of the membrane form of ER (mER)-α and thus address the receptor subform that is involved in these mechanisms. Additionally, we further probe the details of mER-α-mediated activation of AC and the contributions of PDEs to final signal levels, as well as subsequent cAMP-dependent protein kinase A (PKA) activation and the regulation of cell proliferation.

## Methods

### Cell immunoseparation and subculturing

MCF-7 cells originated from the Michigan Cancer Center. We separated them into two subpopulations by immunopanning [14,15] using C-542 carboxyl-terminal ER-α antibody, provided by Drs Dean Edwards and Nancy Weigel. This antibody is now commercially available from Stressgen Biotechnologies (Victoria, Canada). Briefly, sterile antibody on the surface of a petri plate bound cells at 4°C over a 1-hour time period, and cells that attached to the plate (mER^+^) were propagated separately from those that did not bind (mER^low^). Using the same antibody, mER^+ ^cells were then subjected to further separation via a fluorescence-activated cell sorter [16]. Cells selected by both types of sequential separation are highly enriched for mER-α (mER^high^). Upon propagation, the separated cells were not exclusively mER positive or negative, and consequently they were named mER^high ^and mER^low^. We previously observed this heterogeneity in similarly separated GH3/B6 cells [15,17]. All subpopulations of MCF-7 cells were routinely cultured in phenol-red free Dulbecco's modified eagle medium (Gibco-BRL, Grand Island, NY, USA) supplemented with 10% heat-inactivated DSCS (defined/supplemented bovine calf serum; HyClone Laboratories, Inc., Logan, UT, USA) and 1% antibiotic–antimycotic (Gibco Invitrogen Corporation, #15240-062). Cells used in the experiments were between passage 8 and 14 after separation.

Three days before each experiment, the cell growth medium was replaced with medium containing 4 × dextran-coated charcoal stripped serum (DCSS medium). Stripped serum was produced by incubation with an equivalent volume of packed 0.25% weight/vol charcoal Norit A for 2 hours on a shaker at 4°C. The charcoal preparation had previously been suspended in 0.0025% (weight/vol) dextran T-70 (Sigma, St Louis, MO, USA) in 0.25 mol/l sucrose, 1.5 mmol/l MgCl_2_, and 10 mmol/l HEPES (pH 7.6). The charcoal was pelleted at 500 *g *for 10 min and the supernatant (stripped serum) decanted, and the process was repeated four times. In other experiments we deprived cells of serum 2–3 days before the experiment by placing them in a defined medium (DM) consisting of phenol-red free Dulbecco's modified eagle medium with 5 μg/ml insulin, 5 μg/ml transferrin, 5 ng/ml selenium (using the stock preparation from Sigma), and 0.1% bovine serum albumin.

### cAMP response measurement

Cells were plated at a density of 0.25 × 10^6 ^per well in six-well plates and then incubated in either DM or DCSS for 3 days before the experiment. In experiments in which hormone treatments were performed at 37°C, on the day of the experiment the cells were pretreated for 10 min with 1 mmol/l IBMX (3-isobutyl-1-methylxanthine; Sigma) and then treated for different time intervals (3, 6, 10, 20, 30, 60 or 120 min) with 1 pmol/l E_2 _or ethanol vehicle (0.1%) in the presence of IBMX. In experiments in which the hormone treatment (as described above) was performed at 4°C, cells were treated either in their growth plates or in suspension. For cell suspensions the cells were scraped from two 150 mm plates, pelleted at 1000 *g*, and resuspended in 3.5 ml DM. The incubation was then performed in 100 μl of cell suspension with permanent agitation and the reaction was terminated by spinning down the cells and washing them in cold DM. For attached cells, similar incubations were performed in the growth dishes.

The specificity of this response for ER-α was tested with ICI172,780 (Tocris Cookson Inc., Ellisville, MO, USA). The cells were pretreated with ICI172,780 for 30 min (1 μmol/l) and incubated for an additional 15 min after adding 1 pmol/l E_2_, or they were simultaneously treated for 15 min with E_2 _and ICI172,780. As a negative control, we also tested the time-dependent production of cAMP with 10 nmol/l 17α-estradiol (Sigma Aldrich, St Louis, MO, USA) – an inactive E_2 _stereoisomer. Preparation of cell lysates and quantification of intracellular cAMP were performed according to the protocol provided with the cAMP detection kit (Amersham Pharmacia Biotech, Piscataway, NJ, USA). Each value for cAMP was normalized against protein concentration determined using a Bradford (Bio Rad, Hercules, CA, USA) protein detection kit.

### Cell proliferation

Cells were plated at a density of 1000 or 2000 cells per well in 96-well plates. The next day the growth medium was replaced with DCSS containing the hormone (E_2_) or other treatments (forskolin, 8-CPT cAMP, H-89 plus E_2_, SQ22 536 plus E_2_). Controls for E_2 _treatment were done on a separate plate, because we had previously determined that low amounts of volatilized estrogens can affect responses mediated via nongenomic signaling pathways [18]; control treatments were with the vehicle in which test compounds were solubilized (ethanol or dimethyl sulfoxide). In some experiments the growth medium was replenished every second day, and after 5 days the cells were fixed with 2% paraformaldehyde/0.1% glutaraldehyde in PBS. The number of the cells in each well was determined using the crystal violet (CV) assay [19], which we modified previously [17]. Briefly, the cells were incubated in 0.1% CV for 30 min at room temperature, excess dye was removed by three brief rinses with ddH_2_O, the plates were air dried, and the dye was extracted with 10% acetic acid, which was then read in a plate reader (Wallac 1420; Perkin Elmer, Boston, MA, USA) at 590 nm.

**Figure 1:**
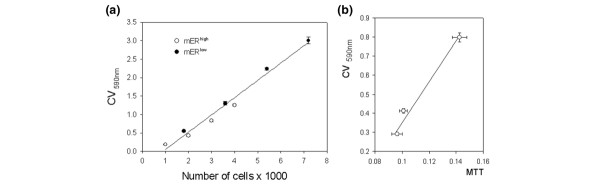
Verification of the utility of the crystal violet (CV) assay for measuring cell number. **(a) **Different numbers of MCF-7 cells enriched for membrane estrogen receptor-α (mER^high^) and MCF-7 cells depleted for membrane estrogen receptor-α (mER^low^; 1000–7000/well) were plated, fixed with 2% paraformaldehyde/0.1% glutaraldehyde, and then quantified via the CV assay at 590 nm absorbance. **(b) **Different numbers of mER^high ^cells were assessed in parallel with CV and MTT assays to demonstrate a linear correlation between these assays. The values are means for 28 samples ± standard error.

The utility of the CV assay in measuring cell number was verified both in MCF-7 cells and in combination with immunodetection plate assays for GH3/B6 cells [17]. We compared CV results with DNA content measurements and with cell number counts by hemocytometer and both assays correlated very well (data not shown). Additionally, we compared the CV method with the MTT assay (ATCC, Manassas, VA, USA), which is often used to determine cell number and viability. Different numbers of mER^high ^and mER^low ^cells (1000–7000) were plated in 96-well plates, and fixed and treated with CV as described. Both sets of data were approximated with a single linear regression line (Fig. 1a). The number of cells determined by CV versus MTT assays were linearly correlated (Fig. 1b).

### Statistical analysis

Statistical differences between two sets of data were determined using two-way analysis of variance. The dose–response curves were fitted with a four-parameter Gaussian distribution (Sigma Plot 8.0; Systat Software Inc., Point Richmond, CA, USA). The differences between the entire curves were tested by comparing the sum of squares of the residuals from each individual curve with the sum of squares of the residuals of the combined curves by applying a Microsoft Excel F test. *P *< 0.05 was considered statistically significant.

## Results

### Optimal conditions for E_2_-induced cAMP accumulation and measurement in MCF-7 cells enriched and depleted for membrane ER-α

#### MCF-7 cell line enriched for membrane ER-α

**Figure 2:**
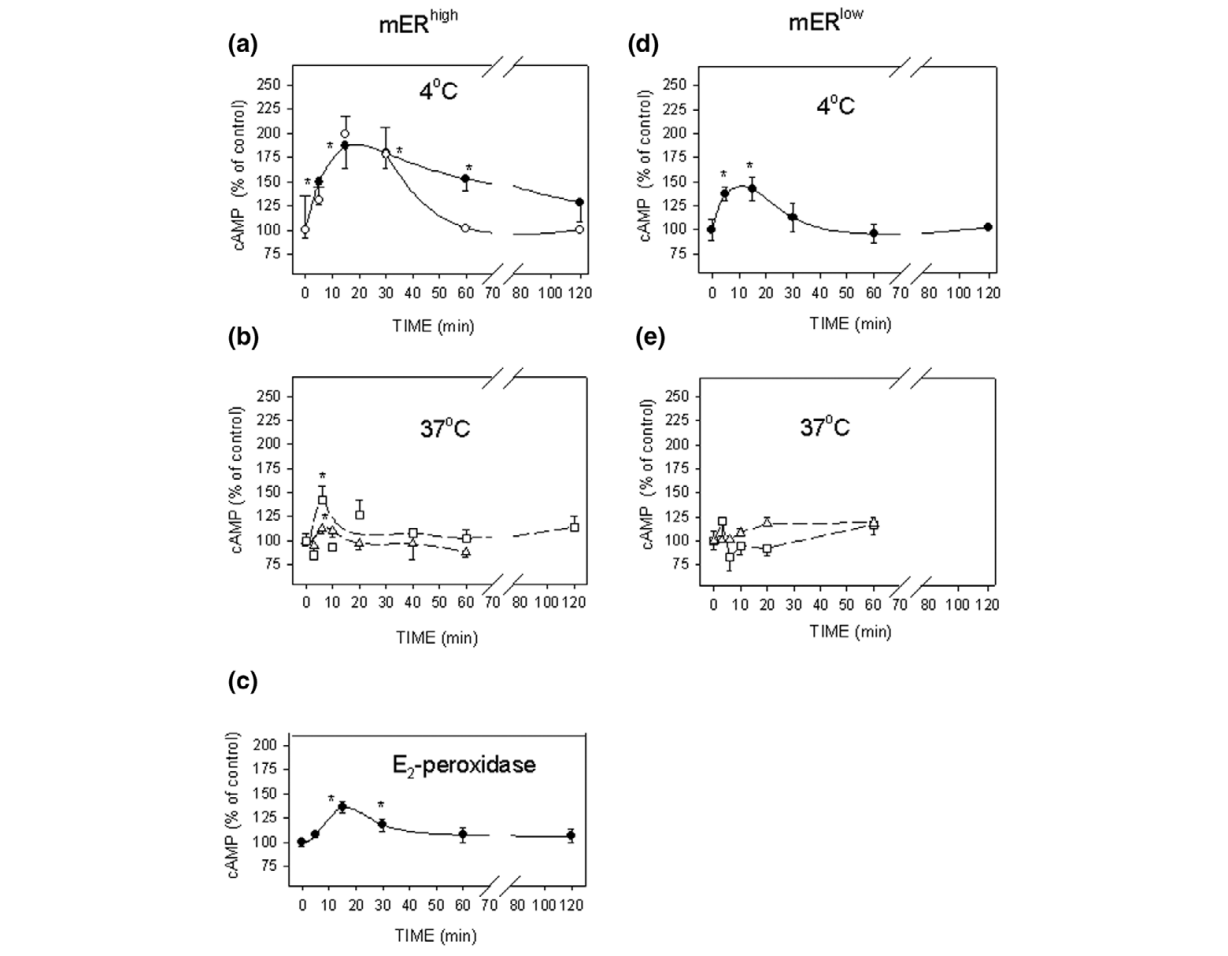
Optimization of conditions for 17β-estradiol (E_2_)-induced cAMP accumulation and measurement. **(a–c) **MCF-7 cells enriched for membrane estrogen receptor-α (mER^high^) and **(d, e) **MCF-7 cells depleted for membrane estrogen receptor-α (mER^low^). All of the cells were stimulated with 1 pmol/l E_2_, or an equivalent amount of E_2 _conjugated to peroxidase, for different time intervals, and the intracellular cAMP levels were assessed. (Panels a and d) Cells were incubated at 4°C in defined medium (DM) either attached to a plate (open circles) or in suspension (closed circles). (Panels b and e) Attached cells were incubated at 37°C in DM medium (triangles) or DCSS medium (medium with 4 × dextran-coated charcoal-stripped serum; squares). (Panel c) Cells in suspension were stimulated with E_2_-peroxidase at 4°C. All experiments were repeated at least three times, and each time point was in triplicate. The data are presented as means ± standard error and the asterisks represent significant differences (*P *< 0.05) as compared with time 0.

We observed that the level and kinetics of cAMP accumulation varied depending on the type of incubation medium, the incubation temperature, and pretreatment with the PDE inhibitor IBMX. In mER^high ^MCF-7 cells, 1 pmol/l E_2 _induced rapid and transient production of cAMP (Fig. 2a,2b,2c). At the reduced temperature of 4°C in a completely defined medium (Fig. 2a), a substantial increase in accumulated cAMP was seen as compared with the levels achieved at 37°C (Fig. 2b). The response peak at 4°C was prolonged, as the accumulated cAMP decreased gradually, even in the absence of the PDE inhibitor IBMX, which is usually included to inhibit the decay of cAMP and enhance the response. The same level of accumulated cAMP was obtained at 5, 15 and 30 min at 4°C, regardless of whether the treatment was performed with cells attached to a plate (Fig. 2a, open circles) or in suspension (Fig 2a, closed circles). However, after 30 min the cAMP level declined abruptly in attached cells. The DCSS medium did not increase cAMP production at 4°C (data not shown).

At 37°C, a low but significant degree of cAMP elevation was achieved by 5 min in attached cells treated in DM (Fig. 2b, triangles); a greater cAMP response was achieved in attached cells pre-incubated in DCSS (Fig. 2b, squares) as compared with DM. All of the experiments conducted at 37°C were performed in the presence of 1 mmol/l IBMX, as in most published protocols.

The impeded ligand E_2_-peroxidase also triggered production of cAMP in suspended cells treated at 4°C in DM (Fig. 2c). The level of E_2 _presented by the conjugate in these studies approximated the level applied as free steroid in the experiments shown in the other panels. The resulting maximal level of accumulated cAMP was lower than that with the equivalent amount of free ligand, but the time required for peak accumulation was the same.

#### MCF-7 cell line depleted for membrane ER-α

When treated at 4°C in suspension, mER^low ^MCF-7 cells exhibited only a small but significant rise in cAMP (Fig. 2d) in response to E_2_. Hormone treatment caused a maximal accumulation of cAMP at 6–15 min, but the decay in this second messenger was very rapid. Insignificant changes were observed at 37°C in attached cells, both in serum-containing and in defined media (Fig. 2e). The statistical comparison of cAMP accumulated in mER^high ^versus mER^low ^cells revealed that those differences were significant.

### cAMP dynamics

**Figure 3:**
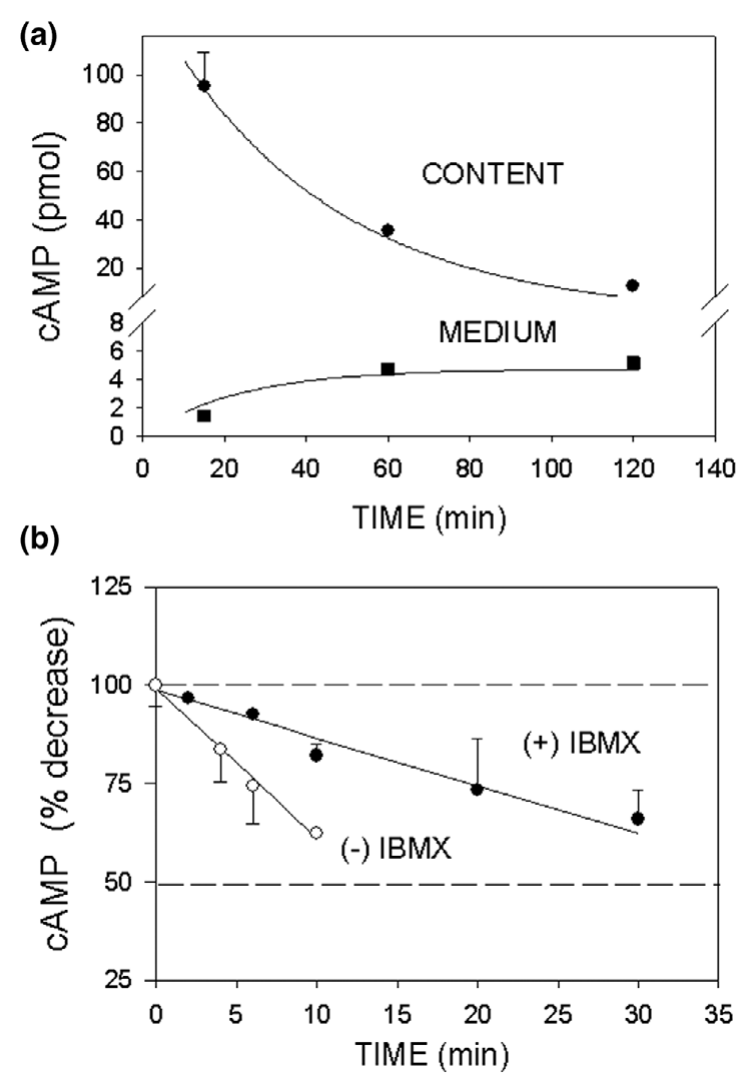
Kinetics of cAMP decrease in MCF-7 cells enriched for membrane estrogen receptor-α (mER^high^) cells. **(a) **Cells were treated with 1 pmol/l 17β-estradiol (E_2_) for different time intervals at 37°C in the presence of 1 mmol/l 3-isobutyl-1-methylxanthine (IBMX). The intracellular cAMP (circles) and that in the medium (squares) from the same cells were assessed. **(b) **cAMP was produced by directly stimulating adenylyl cyclase with 10 μmol/l forskolin for 15 min at 37°C. The decrease in cAMP in the cytosol was tested at 37°C in the absence (open circles) and presence of 1 mmol/l IBMX (closed circles). The entire regression lines were compared by evaluating the differences between the sums of squares of the residuals of individual lines with the sum of squares of the residuals of the combined line using an F-test. The data are presented as means ± standard error. The regression lines were significantly different (*P *= 0.0001).

Because our 4°C protocol without any added IBMX is relatively unusual, we wondered about the causes of the cAMP level dynamics. We next asked why accumulated cAMP did not plateau at 37°C. That is, what are the circumstances of cAMP decay in these cells, and does cAMP leave the cells or is it metabolized? To approach these questions we measured cAMP levels at 37°C after 1 pmol/l E_2 _stimulation, in cells and in their extracellular medium in parallel. We also measured the kinetics of IBMX protection of cAMP by measuring the decrease in cAMP in the cytosolic fraction prepared from cells that had been treated with forskolin. The increase in cAMP in the medium did not match the decrease in intracellular cAMP content of the cell; although the intracellular cAMP of this population of cells decreased by about 80 pmol (from 95 pmol down to 15 pmol), the cAMP in the medium increased only by about 3 pmol (Fig. 3a). As expected, in the absence of IBMX the decrease in cytosolic cAMP was rapid (3.8%/min), whereas in the presence of IBMX it decreased at a slower rate (1.2%/min). Therefore, the addition of IBMX protected cAMP from the action of some PDEs (Fig. 3b), but there was still a significant fraction of cAMP that seemed refractory to this protection.

### ICI172,780 and 17α-estradiol effects on cAMP production

**Figure 4:**
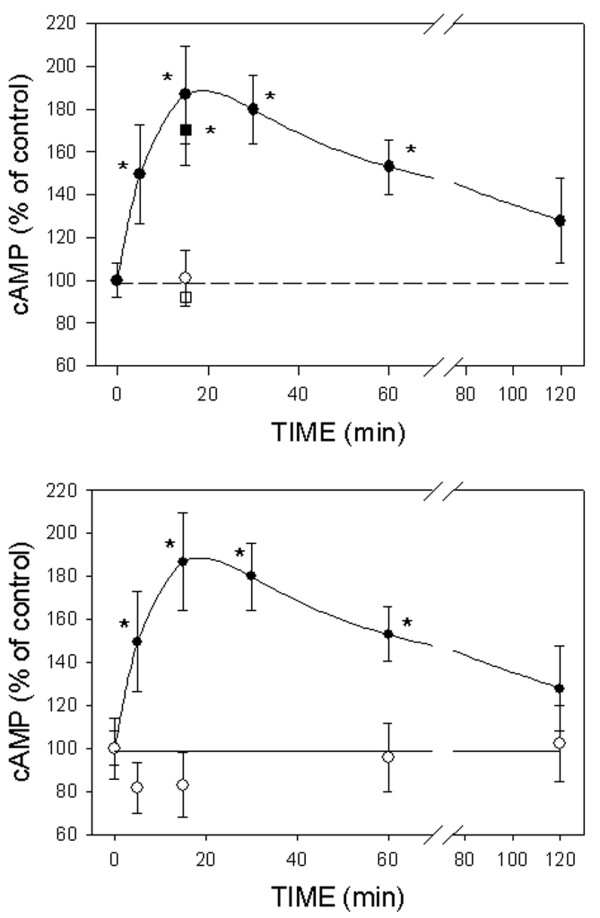
Effects of ICI172,780 and 17α-estradiol on cAMP production in MCF-7 cells enriched for membrane estrogen receptor-α (mER^high^) cells. **(a) **ICI172,780 inhibited 17β-estradiol (E_2_)-induced cAMP production. Both simultaneous application of 1 μmol/l ICI172,780 and 1 pmol/l E_2 _(open circle) or 30 min pretreatment with ICI172,780 (open square) was tested. **(b) **17α-Estradiol was applied at a 10 nmol/l concentration and the time dependent cAMP production was followed. Closed circles represent E_2 _induced cAMP production, redrawn for comparison from Fig. 2a.

ICI172,780 was effective in preventing the 15 min maximal cAMP accumulation (Fig. 4a). Simultaneous application of ICI172,780 (1 μmol/l) and E_2 _(1 pmol/l) was as effective as the 30 min pretreatment with ICI172,780. The usually inactive E_2 _stereoisomer 17α-estradiol (10 nmol/l) was not capable of triggering cAMP production in mER^high ^MCF-7 cells (Fig. 4b). These studies are consistent with this effect being mediated by known ER proteins.

### E_2_-induced dose-responses of cAMP levels and cell number changes differ between MCF-7 cells enriched and depleted for membrane ER-α

**Figure 5:**
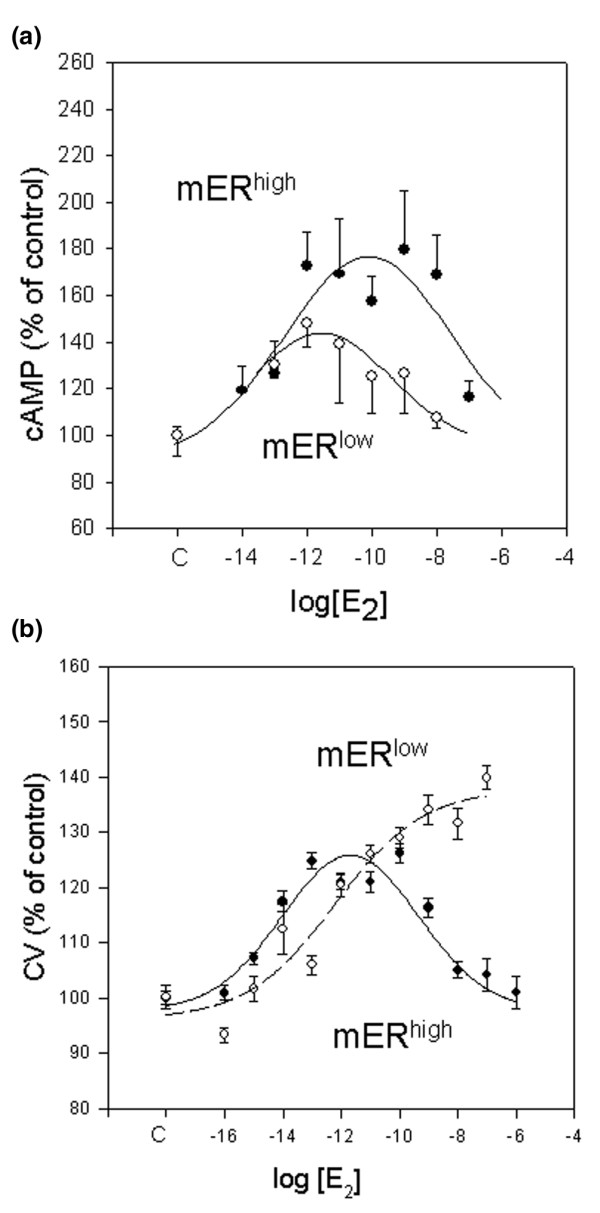
17β-Estradiol (E_2_) dose-dependent cAMP responses and proliferation in MCF-7 cells enriched for membrane estrogen receptor-α (mER^high^; closed circles) versus MCF-7 cells depleted for membrane estrogen receptor-α (mER^low^; open circles). **(a) **cAMP response; each experiment was repeated three times with triplicate samples, and the data were approximated with a four-parameters Gaussian equation. The two sets of data were compared by evaluating the differences between the sums of squares of the residuals from each individual curve with the sum of squares of the residuals of the combined curve using the F-test. The curves were significantly different (*P *= 0.02). **(b) **E_2 _dose-dependent proliferation curves vary with mER expression level. mER^high ^cells (closed circles) exhibited a biphasic growth pattern, whereas mER^low ^cells (open circles) responded with a sigmoid growth pattern. The cells were treated on separate plates with different concentrations of E_2 _or ethanol vehicle (0.1%) for 5 days. The data are percentages of control, and expressed as mean values ± standard error from three separate experiments, each containing 40 replicates.

In Fig. 2 we observed that the characteristics of hormonally induced cAMP accumulation differed between mER^high ^and mER^low ^cells. Figure 5a shows that the E_2 _dose–response curve for this response was biphasic in both cell types, but the sensitivity and upper limit of the responses differed. The mER^high ^cells exhibited a higher maximal response level in cAMP levels. In mER^low ^cells the response peak was achieved with a 50-fold lower E_2 _concentration; the higher maximal stimulation of cAMP production in mER^high ^cells was achieved with 100 pmol/l E_2_. Therefore, it can be assumed that a larger number of receptors had to be filled to achieve this higher response level in cells that had a larger receptor pool.

E_2 _also caused different dose effects on the proliferation of mER^high ^versus mER^low ^cells. Cells with low mER levels responded to E_2 _with stimulated proliferation in the whole range of tested concentrations (from 0.1 fmol/l up to 0.1 mmol/l; Fig. 5b, open circles). However, mER^high ^cells showed a biphasic proliferation pattern, with growth stimulation in the lowest range of concentrations of E_2 _(up to 1 pmol/l) and inhibited proliferation at higher E_2 _concentrations (from 1 nmol/l up to 1 mmol/l; Fig. 5b, closed circles). Therefore, the higher levels of cAMP production achieved with mER^high ^cells result in growth inhibition.

**Figure 6:**
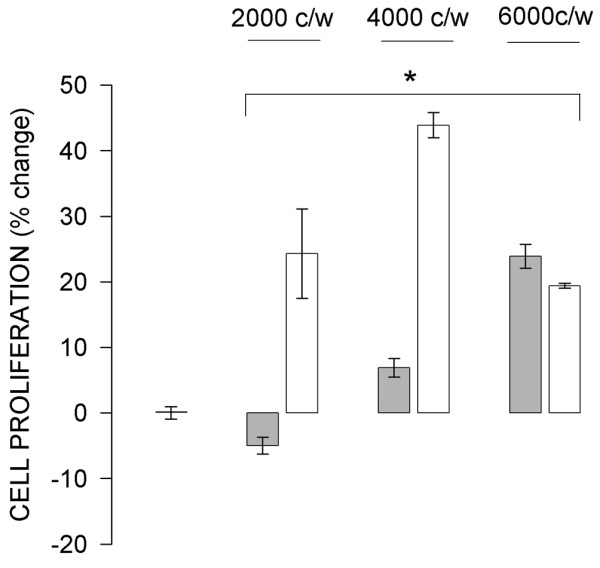
Effect of cell density on 17β-estradiol (E_2_)-induced proliferation. Different densities of MCF-7 cells enriched for membrane estrogen receptor-α (mER^high^; shaded bars) and MCF-7 cells depleted for membrane estrogen receptor-α (mER^low^; open bars) were treated for 5 days with 10 nmol/l E_2_. The controls were treated on a separate plate with ethanol vehicle (0.1%). Values are expressed as percentage change from control. All experimental mean values (error bars are ± standard error) were significantly different (*P *< 0.002) from the control (first bar on the left).

Because we had previously observed an effect of cell density on the level of mER expression and corresponding functional consequences in pituitary tumor cells, we wondered whether cell density would affect functional responses to E_2 _in this breast cancer model system. Indeed, proliferation of mER^high ^cells in the presence of 10 nmol/l E_2 _was influenced by cell density (Fig. 6). At a low density (such as that used in the preceding experiments) proliferation was inhibited, whereas at higher cell densities this effect was reversed. When the cells were plated at the highest density studied (6000/well), their growth was stimulated at the same level as were the mER^low ^cells. In contrast, proliferation of mER^low ^cells was stimulated regardless of the cell plating density.

### E_2_-induced increase in adenylyl cyclase activity correlates with decreased proliferation of MCF-7 cells enriched for membrane ER-α cells via a PKA-activated pathway

**Figure 7:**
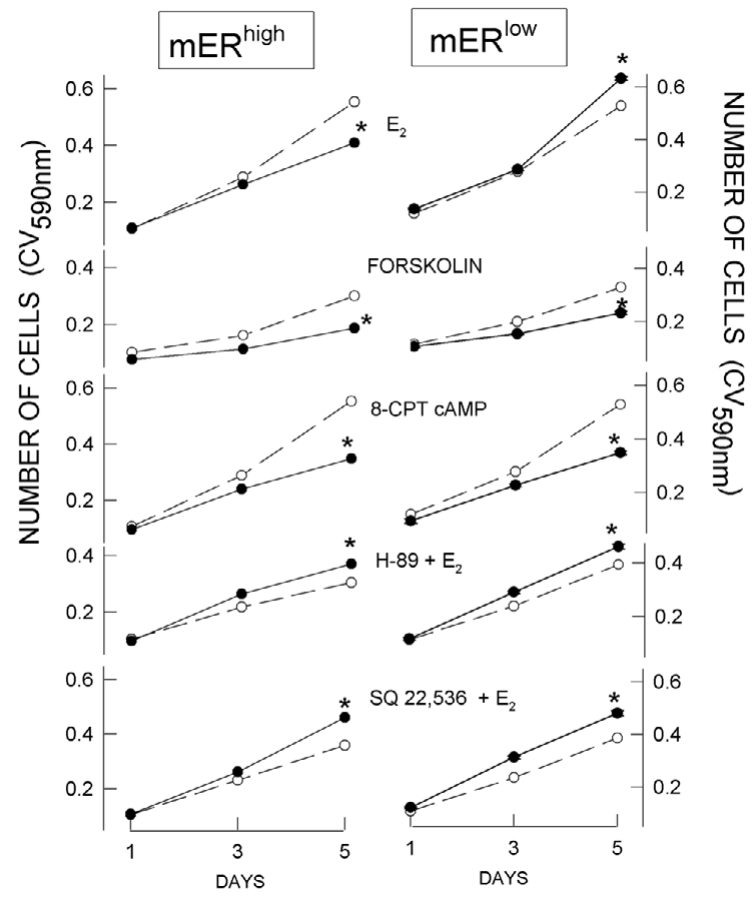
Signaling pathway inhibitors differentially affect cell proliferation. 17β-Estradiol (E_2_; 10 nmol/l) was introduced on day 0. Adenylyl cyclase stimulator (forskolin; 10 μmol/l) and cAMP analog (8-CPT cAMP; 250 μmol/l) were replenished every second day. PKA inhibitor (H-89; 10 μmol/l) and adenylyl cyclase inhibitor (SQ 22,536; 300 μmol/l) were applied at day 0 together with 10 nmol/l E_2_. Cells were fixed on days 1, 3 and 5, and their number estimated by crystal violet (CV) assay. The data are averaged values ± standard error (error ranges were very small and are contained within the size of the symbol) of two separate experiments, each containing 24 replicates. mER^high^, MCF-7 cells enriched for membrane estrogen receptor-α; mER^low^, MCF-7 cells depleted for membrane estrogen receptor-α.

**Figure 8:**
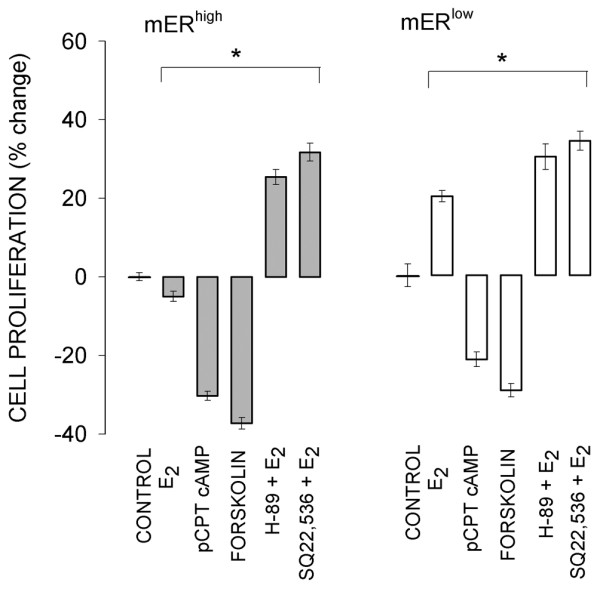
Proliferation of cells after 5 days of treatment with different compounds. The same incubation conditions apply as for Fig. 7. The asterisks indicate significant differences (*P *< 0.002) from controls. Data are expresssed as means ± standard error. E_2_, 17β-estradiol; mER^high^, MCF-7 cells enriched for membrane estrogen receptor-α; mER^low^, MCF-7 cells depleted for membrane estrogen receptor-α.

An activator of AC (forskolin), as well as the cell-permeable cAMP analog 8-CPT cAMP, inhibited the growth of both mER^high ^and mER^low ^cells (Figs 7 and 8) when provided in the same concentrations to both cell types. In mER^high ^cells, both the AC inhibitor SQ22,536 and the PKA inhibitor H-89 abrogated E_2_-induced inhibition of cell proliferation (Figs 7 and 8). Both inhibitors increased the stimulatory effect of E_2 _on mER^low ^cell proliferation (Fig. 8). Therefore, bypassing receptor-mediated signaling mechanisms and controlling cAMP levels with other compounds (which either provided or acted to generate cAMP) made both cell types behave similarly with respect to changes in cell number. Likewise, artificially decreasing cAMP levels or their downstream effects (AC and PKA inhibitors) made cells respond similarly with respect to growth, regardless of their mER levels.

## Discussion

Our studies provide evidence that membrane-associated ERs participate in the estrogen-regulated control of MCF-7 breast cancer cell number by changing cAMP levels and downstream cAMP-activated PKA. E_2 _differentially influenced cell proliferation in mER^high ^versus mER^low ^cells. Measurements of and responses to cAMP manipulated via different culture conditions, assay incubation conditions, and specific response mimetics or inhibitors allowed us to implicate pathways and signal levels in the mechanism of control of cell number, and to suggest possible resolutions between previously reported conflicting results about cAMP and the control of breast cancer cell growth.

Breast cancer cell lines (especially MCF-7 cells) are heterogeneous populations of cells; other investigators have succeeded in separating them into different constituent subpopulations [20,21]. We exploited this characteristic and separated MCF-7 cells into populations enriched and depleted for the membrane subform of ER-α by capturing live cells that bound ER-α-specific antibody to their membranes. These two cell subtypes express the same level of total (membrane plus nuclear) ER [22]. Thus, we have a system in which the consequences of mER levels can be assessed in the same cell line, without other transgenetic manipulations. Because our mER^high ^and mER^low ^cells were not clonally derived (from a single cell), the differences between these populations that we observed can not be attributed to clonal variations.

Attempts to study E_2_-stimulated cAMP levels using the conditions reported by others for MCF-7 cells [23-25] resulted in variable and marginal responses in our hands. We tested different incubation times from 10 min up to 3 hours at 37°C, and all were relatively ineffective, even in the presence of a PDE inhibitor. When we decreased the incubation temperature to 4°C, the amplitude of maximally accumulated cAMP increased and the slope of its decline decreased considerably. Although this temperature is not physiologic, it is often applied for biochemical assessment of biologic responses. This is done to avoid competition by other biologic responses that could rapidly remove the molecule under study at the physiologic temperature.

We also tested a variety of other conditions, including much shorter incubation times than 10 min and various media, as well as comparing attached versus suspended cell preparations. Both we and others [24] have found an influence of charcoal-stripped serum in the medium on cAMP production. However, whereas Fortunati and coworkers [24] could not identify any cAMP production in serum-free medium, we were able to detect a low but significant E_2_-induced increase. It is possible that this discrepancy could be due to different incubation times, as we found a very rapid effect (3 min) but the other study tested 15 min as the earliest time point. In more recent analyses it has become common to observe nongenomic responses that are complete and return to baseline by 15 min [26-28].

In our cells 1 mmol/l IBMX was only partially effective, and loss of cAMP was not the consequence of its transport out of the cells. We can assume that some other PDEs that are resistant to IBMX were present, unless our mER^high ^cells possess other cAMP effectors (receptors or binding proteins) apart from PKA [29] that could capture cyclic nucleotides and render them undetectable in our cytosol assay. The possibility of multiple PDEs with their own individual patterns of regulation provide significant alternative opportunities for control of this important cell function, and add another level of complexity to its analysis.

The impeded ligand that we used in these studies, E_2_-peroxidase, was also capable of triggering cAMP accumulation. The attachment of E_2 _to a large molecule that cannot easily enter cells has previously been used to demonstrate action at the membrane [30-33]. Although this compound stimulated lower but significant levels of cAMP accumulation than did approximately equivalent levels of free steroid, it is likely that steric considerations (such as only partial contact of the large molecule with the cell surface; bound protein obscuring adjacent receptors) may prevent ligand–receptor contacts, or that some other aspect of the ligand–receptor complex configuration is disrupted by ligand tethering. Others have also reported that E_2_-peroxidase has lower affinity for the receptor compared with free ligand [34]. Although it has been reported that steroid can escape conjugates and elicit a response, we removed free steroid in our studies by incubating an aliquot with dextran-coated charcoal under conditions that remove more than 99% of free hormone [35] just prior to application of the impeded ligand. The release of steroid from conjugates happens over a much longer period of time than a rapid response [36].

We have shown that E_2 _can play a dual role in breast cancer cells, both stimulating and inhibiting cell proliferation, depending on its concentration, which is consistent with the findings reported by Lippert and coworkers [37]. We have also shown that the same E_2 _concentration (10 nmol/l) could either prevent or enhance mER^high ^MCF-7 cell growth, depending on cell density. We previously showed that increased cell density can dramatically decrease the fraction of ER-α expressed in the cell membrane (see accompanying reports [18] and [22]), and others have observed similar effects on other membrane receptors [18,38]. The estrogenic responses of mER^low ^cells were not affected by cell density, and their mER levels were already low. We therefore suggest that this type of control can be attributed to effects on the level of mER and thus the signaling cascades elicited by it.

It remains to be determined whether E_2_-induced apoptosis, necrosis, or cell growth arrest could be part of the negative growth effects caused by high E_2 _levels in mER^high ^cells. Others have shown that very low concentrations of E_2 _(as low as 1 pmol/l) induce apoptosis in MCF-7 LTED cells [39], selected by long-term growth in the absence of estrogens. The resulting increase in ER-α expression levels in these cells caused hypersensitivity to E_2 _[40]. In addition, cells in which ER-α has been over-expressed by stable transfection also die in response to physiologic estrogen concentrations [41]. However, measurements in both of these studies failed to distinguish between nuclear and membrane forms of ER-α. Because our mER^high ^cells were selected on the basis of their high mER expression levels (see accompanying report [22]), we can assume that their higher sensitivity to E_2 _is a consequence of their mER levels. Our mER^high ^cells exhibited a biphasic pattern of proliferation, with the maximal growth at approximately 10^-11 ^mol/l E_2 _and the decline in cell number beginning in response to approximately 1 nmol/l E_2_. Maximal stimulation of LTED cells was achieved at 10^-14 ^mol/l, while stimulation of wild-type MCF-7 cells was maximal at 10^-10 ^mol/l E_2_. Hence, our mER^high ^cells exhibit an intermediary proliferation pattern between wild-type and MCF-7 LTED cells with respect to the E_2 _dose–response curves reported by Santen and coworkers [21].

Our mER^low ^cells responded to E_2 _by dose-dependent enhancement of their cell numbers. These cells have much lower levels of mER but some is present, and they express the intracellular ER at comparable levels to mER^high ^cells (see accompanying report [22]). MCF-7 breast cancer cells have also been shown to respond to E_2 _as an apoptosis survival factor after taxol or ultraviolet exposure [42]. This protective effect was directly dependent on the concentration of E_2_, and the highest effect was achieved with 10 nmol/l E_2_. This inhibition of apoptosis was also achieved with E_2_–bovine serum albumin, which indicated probable mediation through a plasma membrane ER. However, these studies did not directly assess the cellular level of mER (the populations studied were probably mixed, comprising cells with both high and low levels of mER). Therefore, it is hard to correlate their heterogeneous cell population results with our selected mER^high ^or mER^low ^cells. Whether protection from apoptosis plays any role in the survival of our mER^low ^cells remains to be determined.

**Figure 9:**
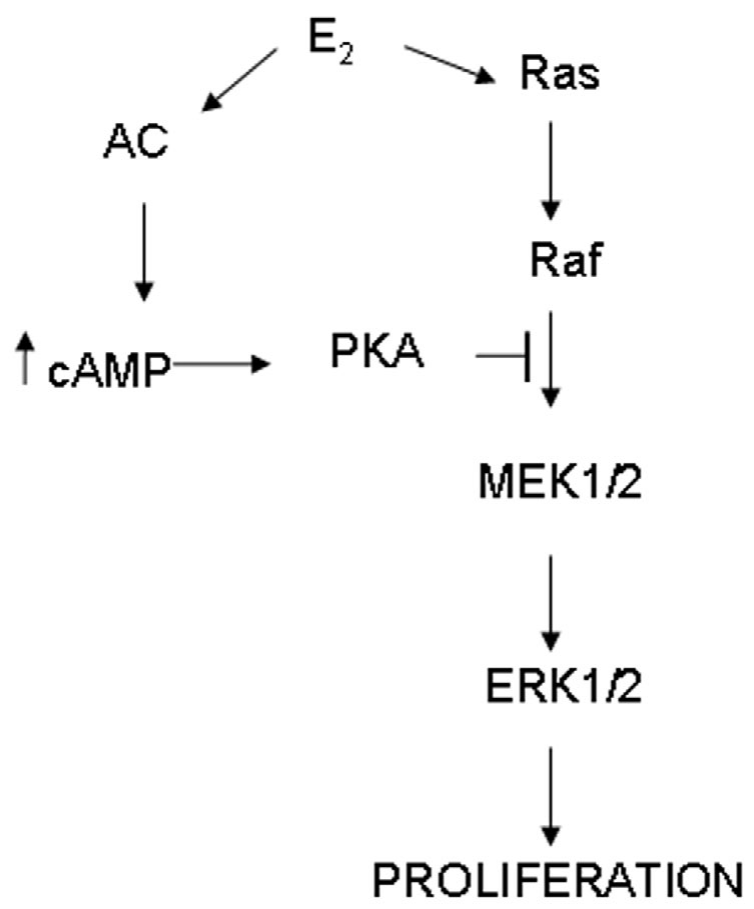
Possible negative feedback effects of estrogen-activated cAMP-PKA (protein kinase A) pathway on the estrogen-activated Ras-Raf-MAPK (mitogen-activated protein kinase) pathway of cell proliferation. The up arrow indicates a cAMP increase. AC, adenylyl cyclase; E_2_, 17β-estradiol; ERK, extracellular signal-regulated kinase; MEK, MAPK kinase.

Our findings also demonstrated that cAMP-activated PKA activity is associated with inhibition of cell proliferation. In mER^high ^cells H-89 blocked the decrease in cell number caused by 10 nmol/l E_2_, and in mER^low ^cells it further enhanced the already existing stimulation of proliferation. It is still not clear how cAMP inhibits cell proliferation. Lowe and coworkers [43] suggested that the inhibitory effect of cAMP (and forskolin, which induced it) occurs distal to extracellular signal-regulated kinase (ERK) activation, possibly by the inhibition of an ERK-independent pathway. However, other groups have reported that the cAMP/PKA pathway and mitogen-activated protein kinase (MAPK) pathway are connected in fibroblasts [43,44] and in MCF-7 cells [45,46]. Activated PKA phosphorylates Raf and inhibits phosphorylation of downstream MEK1/2 and Erk1/2, thus preventing proliferation, a counterbalance to E_2_-stimulated pathways that stimulate proliferation (Fig. 9). Our findings are consistent with the participation of PKA in inhibition of cell proliferation.

In mER^low ^cells, at both low (1 pmol/l) and high (10 nmol/l) E_2 _concentrations, a very low amount of cAMP accumulated. Therefore, in these cells cAMP's inhibitory concentration is never achieved, although further inhibition of cAMP and its downstream effects (via inhibitors) could still enhance proliferation. In mER^high ^cells, however, both low and high E_2 _concentrations caused about the same amount of cAMP accumulation, but only at 10 nmol/l E_2 _did the inhibitory pathway prevail. This finding suggests that the model depicted in Fig. 9 is simplified, and that other pathways participate and integrate at other levels, resulting in modulation of the cell's decision to proliferate, arrest, or die. It was recently shown that E_2 _simultaneously activates the proliferative MAPK-Erk (via Src-Shc-Ras-Raf-MEK-Erk) and phosphatidylinositol-3 kinase pathways in MCF-7 cells, and that both pathways are required to trigger S-phase entry of cells. In addition, the temporal pattern of their activation is extremely important; a necessary immediate and transient consequence of hormone treatment is MAPK activation, whereas phosphatidylinositol-3 kinase activation must be sustained for at least 3 hours [47]. Our results represent a unique example of the extremely complex effects of steroid on target tissues, and emphasize the importance of a global approach when studying the proliferative effects of estrogens.

Reports from other groups have indicated that there may be other membrane estrogen receptor types, such as GPR30 [48] and SHBG-R [24,49], which participate in E_2 _signaling from the membrane. Clearly, a detailed analysis of different mER levels on the same cell backgrounds from a variety of responsive tissue types would be a valuable addition to these investigations. Finally, estrogen-dependent breast cancer cells synthesize and secrete growth factors in response to E_2 _which can stimulate the epidermal growth factor receptor signaling pathway to induce proliferation [50]. Therefore, comparative studies of the contributions of each of these possible players are needed if we are to gain a complete understanding of E_2_-induced proliferation of breast cancer cells.

## Conclusion

Our results indicate that a membrane-associated ER-α is responsible for rapid E_2_-induced cAMP accumulation and subsequent activation of the downstream PKA pathway. A high level of stimulation through the mER-α results in an interruption to breast cancer cell proliferation. Thus, knowledge of tumor mER-α levels and manipulation of estrogenic compound doses and resulting signaling mechanisms may offer an opportunity to refine treatment strategies for some patients with tumors that have these characteristics.

## Abbreviations

AC = adenylyl cyclase; CV = crystal violet; DCSS = medium with 4 × dextran-coated charcoal-stripped serum; DM = defined medium; E_2 _= 17β-estradiol; ER = estrogen receptor; ERK = extracellular signal-regulated kinase; IBMX = 3-isobutyl-1-methylxanthine; MAPK = mitogen-activated protein kinase; mER = membrane estrogen receptor; PDE = phosphodiesterase; PKA = protein kinase A.

## Competing interests

The author(s) declare that they have no competing interests.
